# The impact of three carbapenems at a single-day dose on intestinal colonization resistance against carbapenem-resistant *Klebsiella pneumoniae*

**DOI:** 10.1128/msphere.00479-23

**Published:** 2023-11-27

**Authors:** Huan Kuang, Yongqiang Yang, Huan Luo, Xiaoju Lv

**Affiliations:** 1 Center of Infectious Diseases, West China Hospital, Sichuan University, Chengdu, China; 2 Division of Infectious Diseases, State Key Laboratory of Biotherapy, Chengdu, China; 3 Center for Pathogen Research, West China Hospital, Sichuan University, Chengdu, China; Antimicrobial Development Specialists, LLC, Nyack, New York, USA

**Keywords:** carbapenem, intestinal microbiota, colonization resistance, CRKP, 16S rRNA

## Abstract

Gut microbiota plays a crucial role in providing colonization resistance against multi-drug resistant organisms including carbapenem-resistant *Klebsiella pneumoniae* (CRKP). However, the impact of different carbapenems on intestinal colonization resistance against CRKP remains poorly understood. In this study, we aimed to investigate the effects of three commonly used carbapenems (meropenem, imipenem, and ertapenem) administered at single-day doses, which are typically employed in emergency departments for managing infected patients, on CRKP gut colonization. We conducted experiments in mice by intravenously administering each carbapenem using human-simulated one-day regimens. The composition of the gut microbiota was analyzed using 16S rRNA amplicon sequencing before and after carbapenem administration. Our results revealed that all three carbapenems, when administered at a single-day dose significantly altered the abundance and diversity of the gut microbiota, leading to compromised colonization resistance against CRKP. Notably, the ertapenem and imipenem groups showed an increase in *Allobaculum*, *Bifidobacterium*, *Enterobacteriaceae*, while the meropenem and imipenem groups exhibited a decrease in *Ruminococcaceae*, S24-7, and *Akkermania*. Additionally, the Shannon index exhibited a negative correlation with both the number of days of CRKP colonization CFUs and the duration of bacteria shedding. Furthermore, the count of CRKP in mice after ertapenem administration on the first day and the duration of CRKP shedding were significantly lower compared to meropenem and imipenem. Predictive metabolic pathway analysis demonstrated that the three carbapenems similarly affected a range of metabolic pathways of gut microflora, including carbohydrate metabolism and vitamin B. Our findings emphasize that ertapenem, with its relatively narrow spectrum, minimizes perturbations of the gut microbiota and has a relatively less impact on gut colonization resistance against CRKP.

**IMPORTANCE:**

The intestinal colonization of carbapenem-resistant *Klebsiella pneumoniae* (CRKP) is an important source of clinical infection. Our research showed that even single-day dose use of carbapenems caused CRKP colonization and continuous bacterial shedding, which reminds clinical doctors to prescribe carbapenems cautiously. Whenever possible, ertapenem should be the preferred choice over other carbapenems especially when the identified or highly suspected pathogens can be effectively targeted by ertapenem.

## INTRODUCTION

Carbapenem-resistant *Klebsiella pneumoniae* (CRKP) is currently a significant clinical challenge, associated with high mortality rates and significant difficulties in medical treatment (1). For CRKP, colonization in the gut is typically an initial step of subsequent invasive infection (2, 3), which is hindered by the normal gut microbiota, known as colonization resistance (4). The use of antimicrobial agents perturbs the gut microbiota, weakening or eliminating colonization resistance and helping CRKP strains to establish colonization (5–7). Carbapenems are broad-spectrum antimicrobial agents commonly used in clinics for the treatment of severe infections due to Gram-negative bacteria. However, carbapenems with a broad spectrum, in particular, with anti-anaerobic activity could have a significant impact on gut microbiota (8). Meropenem, imipenem, and ertapenem are the three major carbapenems in clinical use. Unlike imipenem and meropenem, ertapenem has a long half-life allowing once-daily administration and has limited activity against *Pseudomonas aeruginosa* and *Acinetobacter baumannii*, rending a relatively narrower antimicrobial spectrum (8).

Many critical patients are admitted to hospitals via the emergency department and in resource-limited countries like China, patients may stay and receive treatment in the emergency department for several days before admission to in-hospitals due to the high demand, in particular, to tertiary referral teaching hospitals. According to the antimicrobial stewardship policy in China, clinicians including emergency doctors could prescribe broad-spectrum antimicrobial agents such as carbapenems for up to 24 hours without the need for approval by authorized physicians such as infectious diseases specialists (9). However, it remains largely unclear about the impact of the single-day scheme of a carbapenem on colonization resistance of gut microbiome against CRKP and the differences among the three commonly used carbapenems. To address the knowledge gap, we used murine models to simulate the real condition in the emergency department, where patients receive intravenous single-day doses of the three carbapenems, for evaluating the impact of colonization resistance by gut microbiome against CRKP.

## RESULTS

### A single-day dose of ertapenem compromised colonization resistance of gut microbiota to CRKP at a lower level than that of meropenem and imipenem

We administered three carbapenems, that is, meropenem, imipenem/cilastatin, and ertapenem, to mice via the tail vein at a single-day dose equivalent to that used in humans. The weight of mice was not significantly different between groups. Then we used a CRKP strain, 140731, for intra-gastric colonization by gavage to mice and then measured its colony-forming units (CFUs) of fecal samples in the next day (time point 3, T3) and monitored the duration of its shedding. This CRKP strain was recovered from an in-patient rectal swab for routine surveillance for CRKP and was previously subjected to whole genome sequencing by us (GenBank accession no. JAVBHY000000000.1). This strain belongs to sequence type 11 (ST11) and capsular type KL64, the dominant CRKP type in China (10, 11), and carries *bla*_KPC-2_, a carbapenem-encoding gene.

**Fig 1:**
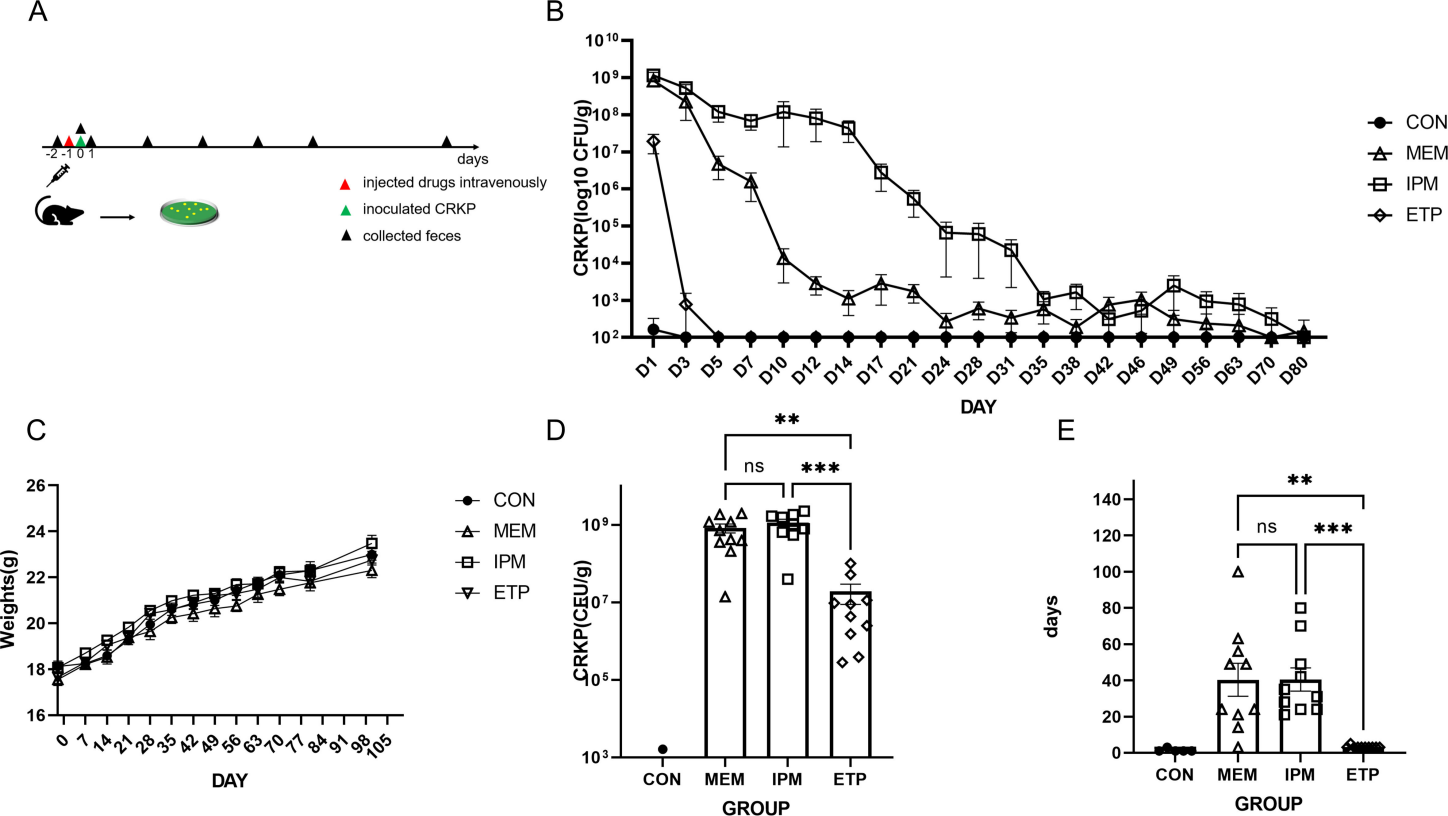
The bacterial count and shedding duration. Panel A, the study process comprising the collection of feces prior to administration with carbapenems on day −2, injection of carbapenems into the tail vein on day −1, collection of feces and then inoculation with CRKP strain on day 0, and subsequent collection of feces. Panel B, a chart showing the trend of bacterial shedding (*n* = 10, mean ± SEM). Panel C, the change in body weight along with the time. Panel D, bacterial on day 1 after inoculation with bacterial strain. Panel E, duration of bacterial shedding in each group. Each dot represents a mouse. Statistical analyses were performed using the Kruskal–Wallis test. Abbreviations: CON, included two groups without administration of a carbapenem nor saline (*n* = 5) and saline (*n* = 5); ETP, etapenem; IPM, imipenem/cilastatin; MEM, meropenem; Error bar, 95% confidence interval (CI). Ns for not significant and *P* > 0.05, * for *P* ≤ 0.05, ** for *P* ≤ 0.01, *** for *P* ≤ 0.001, **** for *P* ≤ 0.0001.

In the saline control group, the duration of bacterial shedding was 0–3 days, indicating that the unperturbed intestinal microbiota indeed confers resistance to CRKP colonization. In the ertapenem group, the duration of bacterial shedding was 3–4 days (Fig. 1E, *n* = 10, mean ± standard error of mean [SEM], 3.2 ± 0.20 days), slightly longer than that in the saline group (*P* = 0.6943, Kruskal-Wallis test that also applied in this section). In contrast, such duration was much longer in the meropenem (Fig. 1E, *n* = 10, 40.30 ± 9.11 days, *P* = 0.0015) or imipenem (Fig. 1E, *n* = 10, 40.40 ± 6.42 days, *P* = 0.0003) group than that in the ertapenem group. Bacterial counts were 3.26 ± 3.26 × 10^2^ CFU/g (*n* = 5, mean ± SEM) in the saline group on the first day after administration of the CRKP strain. In contrast, bacterial counts were 1.91 ± 1.02 × 10^7^ CFU/g in the ertapenem group, higher than that in the saline group (Fig. 1C, *P* = 0.4416) and much lower than that (Fig. 1C, 8.25 ± 2.15 × 10^8^ CFU/g, *P* = 0.0026) in the meropenem group and (Fig. 1C, 11.48 ± 2.39 × 10^8^ CFU/g, *P* = 0.0003) in the imipenem group. Both duration and bacterial counts were not significantly different between meropenem and imipenem groups. We did not find CRKP in the liver, spleen, cecum, and blood of the mice at the end of the experiment after 100 days of CRKP colonization. The above results demonstrated that the use of carbapenems at a single-day dose compromised colonization resistance against CRKP in the gut and among the three carbapenems, ertapenem led to a minor effect on such resistance, much lesser than imipenem and meropenem.

### Each of the three carbapenems at a single-day dose had different impact on gut microbiota composition

**Fig 2:**
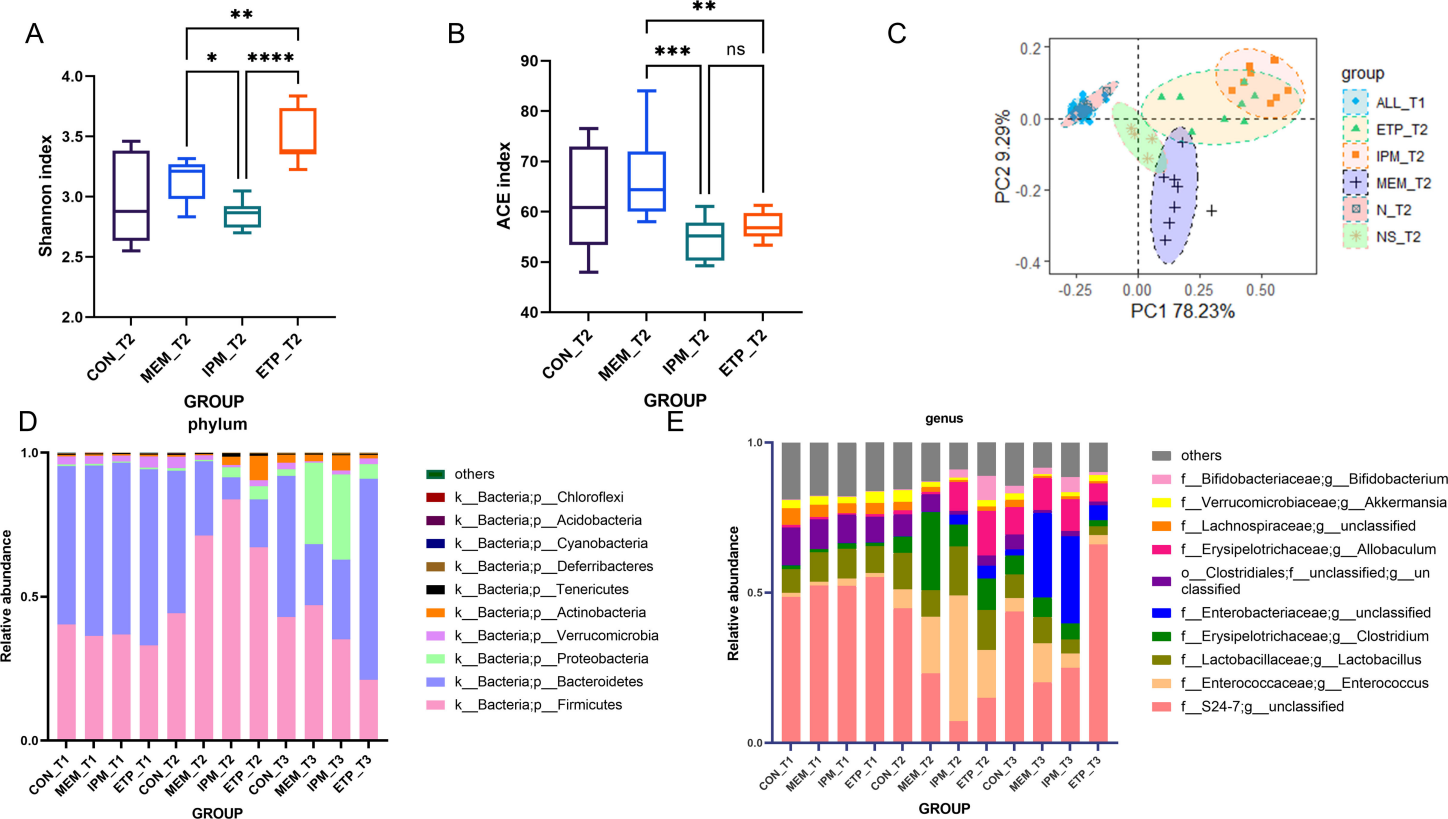
The diversity and richness of murine gut microbiota before and after administration of a carbapenem. Panels A and B, the boxplots of Shannon index and ACE index after administration of a carbapenem (*n* = 8), respectively. Panel C, diversity between murine microbiomes (β diversity) was assessed by principal coordinated analyses (PCoA) (Bray-Crutis matrix) of ASVs and every clustering of before (**T1**) and after (**T2**) administration of a carbapenem (*n* = 8), every ellipse represents the 95% CI of a group. Panels D and E, histograms of the top 10 taxa species abundance at the phylum level and at the genus level, respectively. CON, included two groups without administration of a carbapenem nor saline (*n* = 4) and saline (*n* = 4); ETP, etapenem; IPM, imipenem/cilastatin; MEM, meropenem; N, without administration with a carbapenem nor saline; NS, saline. T1, T2, and T3 are the time points on the day before carbapenem administration (as shown in Fig. 1A day −2), after carbapenem administration on the same day (day −1), and after inoculation of the bacterial strain on the same day (day 1). Ns for not significant and *P* > 0.05, * for *P* ≤ 0.05, ** for *P* ≤ 0.01, *** for *P* ≤ 0.001, **** for *P* ≤ 0.0001.

We collected fecal samples from mice before (time point 1[T1]) and after carbapenem administration (time point 2[T2]) for 16S rRNA amplicon sequencing and subsequent identification of amplicon sequence variants (ASVs). We found that the gut microbial composition was similar with p_*Firmicutes* and p_*Bacteroides* as the dominant phyla (p_ represents a phylum) before treatment across all groups (Fig. 2D). The three carbapenems, despite belonging to the same class, led to distinct changes in the composition of gut microbiota (Fig. 2C). The Shannon index, which reflects the number of species in a population and the evenness of individual distribution among different species, in the ertapenem group (*n* = 8) was significantly higher than those in the meropenem (*n* = 8, *P* = 0.0029, one-way analysis of variance [ANOVA] that also applied in this section) and imipenem groups (*n* = 8, *P <* 0.0001) (Fig. 2A; Table S1). The Shannon index in the meropenem group was also higher than those of imipenem group (*P* = 0.0128). The abundance-based coverage estimator (ACE) index, reflecting the species richness, was also significantly different among the groups (*P* = 0.0006) (Fig. 2B; Table S1). The ACE index in the meropenem group was significantly higher than those of ertapenem group (*P* = 0.0061) and imipenem group (*P* = 0.0008). There was no difference between the imipenem and ertapenem groups (*P* = 0.6533). We analyzed the correlation between the Shannon index of the gut microbiota after administration with carbapenem and CRKP bacterial counts (*r* = −0.6035; 95% confidence interval [CI], –0.8141 to −0.2527; *P* = 0.0018, Spearman correlation) or the duration of CRKP shedding (*r* = −0.6153; 95% CI, –0.8203,–0.2702; *P* = 0.0014, Spearman correlation) that all exhibited a negative correlation.

**Fig 3:**
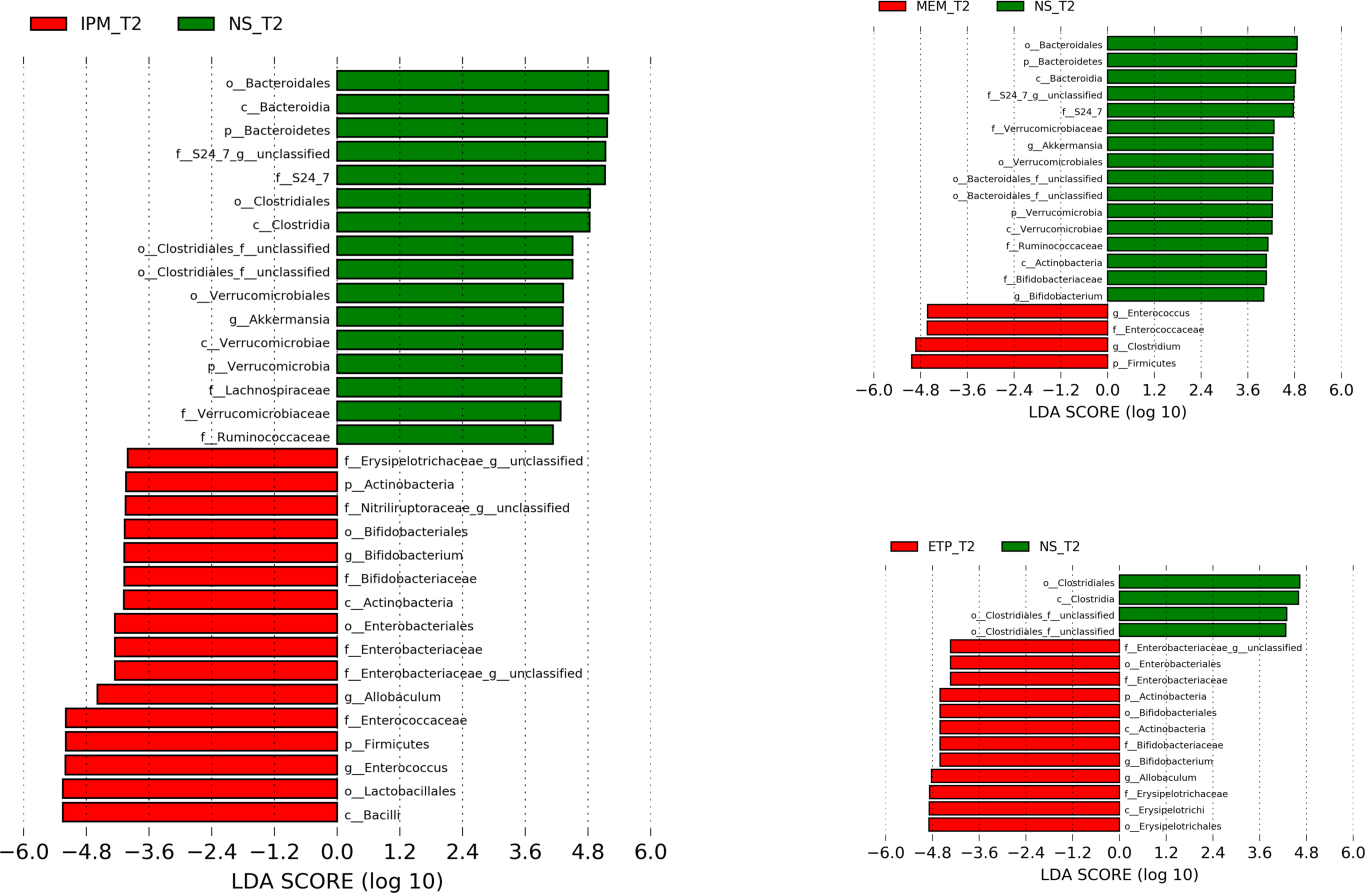
LEfSe for analysis of murine gut microbiota ASVs composition between a carbapenem group and the saline control group after antibiotic or saline treatment (LDA > 4). The length of the bar chart represents the contribution of different species. ETP, etapenem; IPM, imipenem/cilastatin; MEM, meropenem; NS, saline. T2 is the time point on the day after carbapnem administration (as shown in Fig. 1A day −1), and detailed taxonomic labels can be found in Table S2.

We used the Bray-Crutis matrix to conduct principal coordinated analyses (PCoA). The main components of the intestinal microbiota in mice were basically consistent before antibiotic administration, while there were significant differences between the groups after administration (Fig. 2C, D, and E). We also used LEfSe (the linear discriminant analysis effect size, linear discriminate analysis [LDA] > 4) (12) to filter ASVs for differential microbiota between each carbapenem group and the saline group (Fig. 3; Table S2). All three carbapenems caused an increase of p_*Firmicutes*. Specifically, in the ertapenem group, g_*Allobaculum* of p_*Firmicutes* exhibited a notable increase, as did p_*Actinobacteria* (g_*Bifidobacterium*) and p_*Proteobacteria* (f_*Enterobacteriaceae*) (f_ and g_ represent family and genus, respectively). In the meropenem group, p_*Firmicutes* (g_*Enterococcus* and g_*Clostridium*) were the major bacteria increased, while in the imipenem group p_*Actinobacteria* (g_*Bifidobacterium* and f_*Nitrurirutoraceae*), p_*Firmicutes* (g_*Allobaculum* and g_*Enterococcus*), and p_*Proteobacteria* (f_*Enterobacteriaceae*) were increased.

We also observed that the relative abundance of some bacteria was decreased. All three carbapenems led to decrease of bacteria of c_*Clostridia* (c_ represents class) bacteria but each carbapenem affect bacteria of orders or families. In the ertapenem group, o_*Clostridiales* (o_ represents order) was mainly affected. In the meropenem group, f_*Ruminococcaceae* of c_*Clostridia* showed a notable decrease, in addition to p_*Actinobacteria* (g_*Bifidobacterium*), p_*Bacteroides* (f_S24-7), and p_*Verrucomicrobia* (g_*Akkermania*). The imipenem group mainly exhibited a reduction in f_*Ruminococcaceae* and f_*Lachnospiraceae* of c_*Clostridia*, and other bacterial communities such as p_*Bacteroides* (f_S24-7) and p_*Verrucomicrobia* (g_*Akkermania*).

### Each of the three carbapenems at a single-day dose affects different metabolic pathways of the gut microbiota

We predicted the metabolic pathway of murine gut microbiota using PICRUSt2 (13) and compared metabolic pathways between the saline control group and the three carbapenems intervention groups using LEfSe (12) (LDA > 2). Compared with those in the saline control group, the total number of metabolic pathways significantly affected by the ertapenem, meropenem, and imipenem groups was 34, 48, and 77, respectively (Tables S3 and S4). Notably, the number of metabolic pathways affected by ertapenem (*P* < 0.0001, Fisher’s exact test that also applied in this section) and meropenem (*P* = 0.0019) was significantly lower than imipenem, and there was no difference between ertapenem and meropenem (*P* = 0.1020). Nonetheless, all three carbapenems caused changes of certain metabolic pathways in common (Tables S3 and S4). Notably, certain metabolic pathways were reduced that related to carbohydrate metabolism (citrate cycle), glycan biosynthesis and metabolism (N-glycan biosynthesis), metabolism of amino acids (cyanoamino acid metabolism), and metabolism of cofactors and vitamins (riboflavin metabolism, folate biosynthesis, and biotin metabolism). The increased metabolic pathways were mainly carbohydrate metabolism (fructose and mannose metabolism, amino sugar and nucleotide sugar metabolism, propanoate metabolism), amino acid metabolism (lysine biosynthesis), and environmental information processing (phosphotransferase system [PTS]). We also compared the metabolic pathways altered by the meropenem and imipenem groups with those of ertapenem (Table S5) and found that several pathways increased in the ertapenem group including nicotinate and nicotinamide metabolism, selenocompound metabolism, biosynthesis of siderophore group nonribosomal peptides, D-Arginine and D-ornithine metabolism, galactose metabolism, and phenylalanine metabolism. The reduced metabolic pathways were fatty acid biosynthesis, RNA transport, and sulfur relay system.

## DISCUSSION

In this study, we aimed to evaluate the impact of intravenous administration of three carbapenems at a single-day dose, which is commonly used in emergency departments, on the colonization resistance imposed by the gut microbiota in murine models. We found that ertapenem at a single-day dose has minor impact on colonization resistance, much lesser than meropenem and imipenem.

Our study revealed significant changes in the evenness and richness, reflecting by the Shannon and ACE index, respectively, of the gut microbiota after the administration of carbapenems. Ertapenem (14) has a higher intestinal and biliary excretion rate than meropenem (15) and imipenem (16), so this may be the reason for its low ACE index. We found that ertapenem had a notably different impact on the gut microbiota composition from meropenem and imipenem. First, f_*Ruminococcaceae* and f_S24-7 bacteria decreased in both the meropenem and imipenem groups but not in the ertapenem group. The two bacteria can produce short-chain fatty acids (SCFAs) and it has been reported that SCFAs play an important role in the colonization resistance of the gut microbiota against pathogens by coordinating key regulatory factors in the host immune response, regulating intestinal barrier function, or altering the intracellular pH of pathogenic bacteria (17–20). Second, *Akkermansia* spp. decreased in both the meropenem and imipenem groups other than the ertapenem group. *Akkermansia muciniphila* is the only representative of the *Verrucomicrobiota* (21) and has been considered a next-generation probiotic that may have essential functions in maintaining the gut mucosal barrier and regulating immunity (21–23). Third, *Enterococcus* increased in both the meropenem and imipenem groups but not in the ertapenem group. It has been reported (24) that when iron is limited, *Enterococcus faecalis* increases the biofilm formation of *Escherichia coli. E. faecalis* could also promote the growth of co-infecting organisms and multiple microbial infections by overcoming iron limitations (24). It is likely that the increase of *Enterococcus* spp. also contributes to the colonization of *K. pneumoniae* including CRKP.

In animal studies, subcutaneous injection of carbapenem agents has been used to examine the impact on microbiota (6, 25). However, this approach is clearly different from the intravenous injection in clinical use. The administration pathway and pharmacokinetics are important factors that determine the concentration of drugs reaching the intestine (26). Under the research method of subcutaneous injection, it was found that ertapenem did not promote the colonization of extended spectrum-β-lactamase (ESBL)-producing *K. pneumoniae*, while imipenem/cilastatin neither promoted nor inhibited it (25). In clinical studies, compared with a cephalosporin (cefuroxime [27] or ceftriaxone [28, 29]) and metronidazole in combination or piperacillin-tazobactam (29), ertapenem rendered a lower colonization rate of carbapenem-resistant *Enterobacterales* (CREs) (27, 28) and third-generation-cephalosporin-resistant *Enterobacterales* (3GCR-Es) (27, 29). Nonetheless, no studies have been performed to compare the impact of different carbapenems on the intestinal colonization of CRKP.

In addition to perturbing the community structure of the gut microbiota, antimicrobial agents can impair its material metabolism function. Microorganisms in the gut compete with each other and with foreign bacterial strains including pathogens for space and nutrients (30). The observed changes in metabolic pathways, such as riboflavin metabolism (vitamin B2), biotin metabolism (vitamin B7), and folate biosynthesis (vitamin B9) may be linked to the gut immune barrier (31). B group vitamins are crucial intermediates in pathways for many necessary cofactors involved in biological physiological functions, and they are closely related to immune and inflammatory responses (32–35). The increased metabolism of nicotinate and nicotinamide in the ertapenem group could polarize macrophages toward a less inflammatory response to exert anti-inflammatory effects, which may reduce the gut inflammatory response and hinder pathogen colonization (36). The increase in selenocompound metabolism may also facilitate the activation and differentiation of T cells, enhance the bactericidal effect of macrophages, making it difficult for pathogenic bacteria to colonize (37, 38). The microbiota perturbed by ertapenem exhibits an increase in siderophore group non-ribosomal peptides, which may give them a competitive advantage in utilizing iron and limit pro-oxidant mucosal responses, thereby facilitating colonization resistance (39, 40). However, the metabolism of the intestinal microbiota is a complex process, and further studies are needed to confirm the usefulness of the observed metabolic changes in colonization.

We are aware of limitations of this study. First, our study is based on a single-day dose of carbapenems and ertapenem only needs to be administered once daily, while meropenem and imipenem are typically administered three times daily. The difference in the administration scheme introduces a confounding factor for understanding the observed difference between ertapenem and meropenem or imipenem. Nonetheless, the administration schemes in this study are those in clinical use in real world, and therefore the findings here could have the potential to reflect the clinical impact of the three carbapenems. Second, we only sequenced V4 region using high-throughput sequencing amplicon of 16S rRNA for identifying bacteria, which has a relatively low resolution for taxonomic assignations and does not allow accurate identification to the species level. Therefore, in this study, we only performed analysis at the genus level or above.

In summary, our results find that the use of carbapenems even only at a single-day dose could compromise colonization resistance of the gut microbiota against CRKP in murine models. This highlights that carbapenem use should be cautious in clinical settings including the emergency department where antimicrobial agents are typically administered only for one or few days. However, ertapenem at a single-day dose renders much less loss of colonization resistance against CRKP than meropenem and imipenem. Therefore, when a carbapenem is needed, ertapenem should be preferred over other carbapenems if the identified or highly suspected pathogens could be covered by ertapenem. The lesser impact on colonization resistance by ertapenem could be due to the relatively minor alteration of the community structure and metabolisms of the gut microbiota.

## MATERIALS AND METHODS

### Bacterial strain

Strain CRKP140731 was recovered from a patient anal swab for routine surveillance for CRKP and was previously subjected to whole genome sequencing by us (GenBank accession no. JAVBHY000000000.1). This strain belongs to ST11 and KL64, carries *bla*_KPC-2_, and was resistant to carbapenems with MIC of ertapenem, meropenem, and imipenem was 512, 128, and 64 mg/L, respectively.

### Mice and the experiment

Female C57BL/6J mice were purchased from GemPharmatech (Jiangsu, China) at 5 weeks of age. Mice were maintained in a standard animal facility at the Laboratory Animal Center of West China Hospital under SPF conditions with good care. The cage and water are sterilized by autoclaving before use. This study was approved by the Experimental Animal Ethics Committee of the West China Hospital of Sichuan University (registration number: 20211174A).

Mice were randomly divided into four groups (*n* = 5 for each cage, every group two cages) then acclimated for a week in the animal center, they were allowed access to irradiated food and sterile water at will, and a fixed light and dark rhythm was maintained. All mice were weighed and assessed during the study. Carbapenem doses in clinical use, i.e., meropenem 500 mg every 8 h (q8h), imipenem 500 mg q8h, and ertapenem 1 g daily (qd), were calculated and converted to the dose for mice, i.e., meropenem 75 mg/kg q8h, imipenem/cilastatin 75/75 mg/kg q8h, and ertapenem 150 mg/kg qd. The drug is dissolved in 100 µL or 200 µL of physiological saline and injected into the mouse body through the tail vein. The control group was treated with 100 μl of physiological saline (*n* = 5) or non-administration group (*n* = 5). Collect mouse feces once 24 hours after the first administration, and then fresh overnight culture of strain 140731 was orally inoculated into mice at 10^7^ CFU diluted in 200 mL PBS. The time points for collecting mouse fecal sample and CRKP monitoring bacterial shedding are shown in Fig. 1. At different sample collection time points, we collected fecal samples through pressure-induced defecation into 1.5 mL sterile Eppendorf tubes. Two feces per mouse were collected at a time, one sample for 16S rRNA amplicon and another for measuring CRKP burden by mixing with 1 mL of PBS on a vortex. Then, 100 µL homogenized mixture were streaked onto Simmons’ citrate agar plate containing 2 mg/L meropenem. Colonies on plates were counted after 24 hours of incubation at 37°C. Finally, all mice were dissected to evaluate CRKP burden in liver, spleen, cecum, and blood. Tissue samples after weighed were homogenized using tissue grinder (TGrinder H24R, Tiangen, Beijing, China) and 100 µL was inoculated to Simmons’ citrate agar plates for detecting the bacterial growth. Blood (10 µL) was put into 1 mL Luria-Bertani (LB) broth detecting the bacterial growth.

### 16S rRNA amplicon sequencing

Total genomic DNA was extracted from fecal samples by the cetyltrimethylammonium bromide/sodium dodecyl sulfate (CTAB/SDS) method (41). The 16S rRNA of V4 (515F-806R) (42) region was amplified with barcodes. All PCR reactions were performed with Phusion High-Fidelity PCR Master Mix (New England Biolabs, Ipswich, MA, USA). Sequencing libraries were generated using the TruSeq DNA PCR-Free Sample Preparation Kit (Illumina, San Diego, CA, USA), and quantified using the Qubit@2.0 Fluorometer (Carlsbad, CA, USA) and Agilent Bioanalyzer 2100 system (Santa Clara, CA, USA). Thereafter, PCR amplicons were sequenced on an Illumina NovaSeq platform of 250 bp paired-ends.

### Data analysis

The raw reads were assigned to samples based on their unique barcode. Sequence filtering and clustering into ASVs were realized using the q2‐demux plugin dada2 on the QIIME2 platform (version 2020.10) (43, 44). The taxonomic assignation to ASVs was performed using the trained Naïve Bayes classifier which refers to the Greengenes 13_8 99% similarity database (45). The resulted ASVs absolute abundance table and metadata files were used for statistical analysis and data visualization in the R package vegan (46) and ggplot2 (47). PICRUSt2 (13) v2.3.0-b was used to predict the metabolic pathway of the gut microflora and to analyze the metabolic and taxonomic differences between the saline group and the carbapenem group through the LEfSe (LDA >2) (http://huttenhower.sph.harvard.edu/galaxy/) (12). Data and some statistical charts were analyzed using Prism version 9.0, in which one-way ANOVA was used for normally distributed data and Kruskal-Wallis test was used for non-normal distribution data. Fisher’s exact test was used for categorical variable data. *P* < 0.05 were considered statistically significant.

## Data Availability

The data of this study have been deposited into CNGB Sequence Archive (CNSA) (48) of China National GeneBank DataBase (CNGBdb) (49) with accession number CNP0004697.

## SUPPLEMENTAL MATERIAL

Table S1 msphere.00479-23-s0001.pdf Shannon index and ACE index of intestinal microbiota in mice after carbapenem administration.

Table S2 msphere.00479-23-s0002.pdf Compared between each carbapenem group and the saline group to filter ASVs for differential microbiota using LEfSe after antibiotic or saline treatment (LDA > 4).

Table S3 msphere.00479-23-s0003.pdf Compared with the saline group, the carbapenems caused an increase in metabolic pathways.

Table S4 msphere.00479-23-s0004.pdf Compared with the saline group, the carbapenems caused an decrease in metabolic pathways.

Table S5 msphere.00479-23-s0005.pdf The metabolic pathways altered by the meropenem and imipenem groups compared to ertapenem.

ASM does not own the copyrights to Supplemental Material that may be linked to, or accessed through, an article. The authors have granted ASM a non-exclusive, world-wide license to publish the Supplemental Material files. Please contact the corresponding author directly for reuse.
