# Supplementary material for: The impact of three carbapenems at a single-day dose on intestinal colonization resistance against carbapenem-resistant Klebsiella pneumoniae
Source: mSphere. 2023 Nov 27;8(6):e00479-23. doi: 10.1128/msphere.00479-23 (PMC10732052; doi:10.1128/msphere.00479-23)
Supplement: Table S1 — Shannon index and ACE index of intestinal microbiota in mice after carbapenem administration. [file msphere.00479-23-s0001.pdf]

Table S1. Shannon index and ACE index of intestinal microbiota in mice after carbapenems.

samples_ID	Shannon	ACE	group
1A_9	2.5508	48	N_T2
1C_9	2.8786	57.56847	N_T2
1D_9	2.8106	53.37075	N_T2
1E_9	2.6338	60.81548	N_T2
1F_9	3.9966	171.52	NS_T2
1H_9	3.1333	66.31956	NS_T2
1I_9	3.3823	76.51293	NS_T2
1J_9	3.4597	72.96429	NS_T2
2A_9	3.3149	72	MEM_T2
2B_9	2.9717	84	MEM_T2
2C_9	3.2272	59	MEM_T2
2E_9	3.0067	64.35503	MEM_T2
2F_9	3.2724	58	MEM_T2
2G_9	3.2622	72	MEM_T2
2H_9	2.8331	63	MEM_T2
2I_9	3.1954	64.34434	MEM_T2
3A_9	2.9222	56	IPM_T2
3B_9	2.8591	49.21376	IPM_T2
3C_9	2.6992	50.23333	IPM_T2
3D_9	2.8759	50.25	IPM_T2
3E_9	3.0461	57.32585	IPM_T2
3F_9	2.7399	58	IPM_T2
3H_9	2.7565	54.29071	IPM_T2
3J_9	2.922	61	IPM_T2
4B_9	3.3671	55	ETP_T2
4C_9	3.3491	58	ETP_T2
4D_9	3.4517	57.5304	ETP_T2
4E_9	3.3525	61.27106	ETP_T2
4F_9	3.8294	60.29294	ETP_T2
4H_9	3.8371	56	ETP_T2
4I_9	3.3928	53.3488	ETP_T2
4J_9	3.2252	55.30951	ETP_T2

Abbreviations, ETP, etapenem; IPM, imipenem/cilastatin; MEM, meropenem; N, Neither saline nor carbapenems NS; saline. T2 is the time point on the day after carbapnem admisnistation (day -1).
