# Supplementary material for: The impact of three carbapenems at a single-day dose on intestinal colonization resistance against carbapenem-resistant Klebsiella pneumoniae
Source: mSphere. 2023 Nov 27;8(6):e00479-23. doi: 10.1128/msphere.00479-23 (PMC10732052; doi:10.1128/msphere.00479-23)
Supplement: Table S2 — Compared between each carbapenem group and the saline group to filter ASVs for differential microbiota using LEfSe after antibiotic or saline treatment (LDA > 4). [file msphere.00479-23-s0002.pdf]

Table S2. Compared between each carbapenem group and the saline group to filter ASVs for differential microbiota using LEfSe after antibiotic or saline treatment (LDA > 4).

Biomarker_species_names	the logarithm value	increased groups	LDA_values	P_values
k__Bacteria.p__Actinobacteria.c__Actinobacteria.o__Bifidobacteriales.f__Bifidobacteriaceae.g__Bifidobacterium	4.912383353	ETP_T2	4.60788839	0.00658
k__Bacteria.p__Firmicutes.c__Erysipelotrichi.o__Erysipelotrichales.f__Erysipelotrichaceae.g__Allobaculum	5.171306438	ETP_T2	4.82762821	0.00658
k__Bacteria.p__Proteobacteria.c__Gammaproteobacteria.o__Enterobacteriales.f__Enterobacteriaceae_g__unclassified	4.63162028	ETP_T2	4.33250233	0.00658
k__Bacteria.p__Actinobacteria.c__Actinobacteria.o__Bifidobacteriales.f__Bifidobacteriaceae.g__Bifidobacterium	4.439100792	IPM_T2	4.07077087	0.00658
k__Bacteria.p__Actinobacteria.c__Nitriliruptoria.o__Nitriliruptorales.f__Nitriliruptoraceae_g__unclassified	1.946993214	IPM_T2	4.05029128	0.00567
k__Bacteria.p__Firmicutes.c__Erysipelotrichi.o__Erysipelotrichales.f__Erysipelotrichaceae.g__Allobaculum	4.986328788	IPM_T2	4.58955532	0.00658
k__Bacteria.p__Firmicutes.c__Erysipelotrichi.o__Erysipelotrichales.f__Erysipelotrichaceae_g__unclassified	4.561381646	IPM_T2	4.00434352	0.00658
k__Bacteria.p__Proteobacteria.c__Gammaproteobacteria.o__Enterobacteriales.f__Enterobacteriaceae_g__unclassified	4.522487571	IPM_T2	4.24994045	0.00658
k__Bacteria.p__Firmicutes.c__Bacilli.o__Lactobacillales.f__Enterococcaceae.g__Enterococcus	5.273857611	MEM_T2	4.61820756	0.00658
k__Bacteria.p__Firmicutes.c__Erysipelotrichi.o__Erysipelotrichales.f__Erysipelotrichaceae.g__Clostridium	5.414856537	MEM_T2	4.92055836	0.00658

Abbreviations, ETP, etapenem; IPM, imipenem/cilastatin; MEM, meropenem; T2 is the time point on the day after carbapnem admisnistation (day -1).

Table S2. Compared between each carbapenem group and the saline group to filter ASVs for differential microbiota using LEfSe after antibiotic or saline treatment (LDA > 4).

Biomarker_species_names	the logarithm value	decreased groups	LDA_values	P_values
k__Bacteria.p__Firmicutes.c__Clostridia.o__Clostridiales_f__unclassified	4.867797118	ETP_T2	4.2951476	0.00658
k__Bacteria.p__Firmicutes.c__Clostridia.o__Clostridiales_f__unclassified	4.867797118	ETP_T2	4.26755836	0.00658
k__Bacteria.p__Bacteroidetes.c__Bacteroidia.o__Bacteroidales.f__S24_7_g__unclassified	5.540678267	IPM_T2	5.13576237	0.00658
k__Bacteria.p__Firmicutes.c__Clostridia.o__Clostridiales.f__Lachnospiraceae	4.74663278	IPM_T2	4.29526466	0.00658
k__Bacteria.p__Firmicutes.c__Clostridia.o__Clostridiales.f__Ruminococcaceae	4.545294183	IPM_T2	4.1270247	0.00658
k__Bacteria.p__Firmicutes.c__Clostridia.o__Clostridiales_f__unclassified	4.867797118	IPM_T2	4.50540479	0.00658
k__Bacteria.p__Firmicutes.c__Clostridia.o__Clostridiales_f__unclassified	4.867797118	IPM_T2	4.50294734	0.00658
k__Bacteria.p__Verrucomicrobia.c__Verrucomicrobiae.o__Verrucomicrobiales.f__Verrucomicrobiaceae.g__Akkermansia	4.679646658	IPM_T2	4.31951244	0.00658
k__Bacteria.p__Actinobacteria.c__Actinobacteria.o__Bifidobacteriales.f__Bifidobacteriaceae.g__Bifidobacterium	3.568808839	MEM_T2	4.01027624	0.00658
k__Bacteria.p__Bacteroidetes.c__Bacteroidia.o__Bacteroidales.f__S24_7_g__unclassified	5.540678267	MEM_T2	4.78603121	0.00658
k__Bacteria.p__Firmicutes.c__Clostridia.o__Clostridiales.f__Ruminococcaceae	4.545294183	MEM_T2	4.11493802	0.00658
k__Bacteria.p__Verrucomicrobia.c__Verrucomicrobiae.o__Verrucomicrobiales.f__Verrucomicrobiaceae.g__Akkermansia	4.679646658	MEM_T2	4.24810477	0.00658

Abbreviations, ETP, etapenem; IPM, imipenem/cilastatin; MEM, meropenem; T2 is the time point on the day after carbapnem admisnistation (day -1).
