# Supplementary material for: The impact of three carbapenems at a single-day dose on intestinal colonization resistance against carbapenem-resistant Klebsiella pneumoniae
Source: mSphere. 2023 Nov 27;8(6):e00479-23. doi: 10.1128/msphere.00479-23 (PMC10732052; doi:10.1128/msphere.00479-23)
Supplement: Table S3 — Compared with the saline group, the carbapenems caused an increase in metabolic pathways. [file msphere.00479-23-s0003.pdf]

Table S3. Compared with the saline group, the carbapenems caused an increase in metabolic pathways.

Biomarker_KO_ID	Pathway Name	the logarithm value	increased groups	LDA_values	P_values
ko00760	Nicotinate and nicotinamide metabolism	4.032248815	ETP_T2	2.49965407	0.00658
ko05100	Bacterial invasion of epithelial cells	1.421745741	ETP_T2	2.21946205	0.00658
ko02060	Phosphotransferase system (PTS)	4.241963497	ETP_T2	3.38856217	0.00658
ko00450	Selenocompound metabolism	4.02719077	ETP_T2	2.46656422	0.00658
ko00300	Lysine biosynthesis	4.271645634	ETP_T2	2.29406282	0.00658
ko00270	Cysteine and methionine metabolism	4.120427351	ETP_T2	2.86239245	0.00658
ko01053	Biosynthesis of siderophore group nonribosomal peptides	2.650899456	ETP_T2	2.09561002	0.00658
ko00360	Phenylalanine metabolism	3.417233448	ETP_T2	2.53631777	0.00658
ko00362	Benzoate degradation	3.370195916	ETP_T2	2.28077555	0.00658
ko00430	Taurine and hypotaurine metabolism	3.969061271	ETP_T2	2.41675733	0.00658
ko00361	Chlorocyclohexane and chlorobenzene degradation	3.151873756	ETP_T2	2.49905863	0.00658
ko05143	African trypanosomiasis	2.279165861	ETP_T2	2.17738485	0.00658
ko00520	Amino sugar and nucleotide sugar metabolism	4.198262796	ETP_T2	2.91128504	0.00658
ko00052	Galactose metabolism	4.124050976	ETP_T2	2.94893962	0.00658
ko00051	Fructose and mannose metabolism	4.185668695	ETP_T2	3.09354586	0.00658
ko00640	Propanoate metabolism	3.887565672	ETP_T2	2.66403576	0.00658
ko05012	Parkinson disease	0.823920861	ETP_T2	2.51168258	0.00567
ko00472	D-Arginine and D-ornithine metabolism	3.612869296	ETP_T2	3.17324729	0.00658
ko03050	Proteasome	1.98936356	ETP_T2	2.00082015	0.00658

Abbreviations, ETP, etapenem; IPM, imipenem/cilastatin; MEM, meropenem; T2 is the time point on the day after carbapnem admisnistation (day -1).

Table S3. Compared with the saline group, the carbapenems caused an increase in metabolic pathways.

Biomarker_KO_ID	Pathway Name	the logarithm value	increased groups	LDA_values	P_values
ko00564	Glycerophospholipid metabolism	3.814503933	IPM_T2	2.2944725	0.00658
ko00561	Glycerolipid metabolism	3.821359923	IPM_T2	2.60255654	0.00658
ko00010	Glycolysis / Gluconeogenesis	4.257671834	IPM_T2	3.12956119	0.00658
ko05100	Bacterial invasion of epithelial cells	1.288036127	IPM_T2	2.2468008	0.00658
ko02060	Phosphotransferase system (PTS)	4.342303367	IPM_T2	3.66468199	0.00658
ko00300	Lysine biosynthesis	4.274635188	IPM_T2	2.69546518	0.00658
ko03440	Homologous recombination	4.244621865	IPM_T2	2.72215815	0.00658
ko00380	Tryptophan metabolism	3.386301232	IPM_T2	2.2988744	0.00658
ko00627	Aminobenzoate degradation	3.334439581	IPM_T2	2.58663557	0.00658
ko00621	Dioxin degradation	3.769178937	IPM_T2	3.07678115	0.00658
ko00620	Pyruvate metabolism	4.160819445	IPM_T2	2.98205657	0.00658
ko00072	Synthesis and degradation of ketone bodies	4.003167342	IPM_T2	3.23079021	0.00658
ko00362	Benzoate degradation	3.378737994	IPM_T2	2.25723067	0.00658
ko00430	Taurine and hypotaurine metabolism	3.990449102	IPM_T2	2.55735267	0.00658
ko02010	ABC transporters	3.877316572	IPM_T2	2.82117979	0.00658
ko00361	Chlorocyclohexane and chlorobenzene degradation	3.276804932	IPM_T2	2.7346033	0.00658
ko03018	RNA degradation	3.828048711	IPM_T2	2.07127821	0.00658
ko00520	Amino sugar and nucleotide sugar metabolism	4.22635126	IPM_T2	3.10557704	0.00658
ko00053	Ascorbate and aldarate metabolism	3.494532605	IPM_T2	2.62836498	0.00658
ko00051	Fructose and mannose metabolism	4.21454354	IPM_T2	3.18257482	0.00658
ko00480	Glutathione metabolism	3.802787011	IPM_T2	2.72731649	0.00658
ko00640	Propanoate metabolism	3.895104446	IPM_T2	2.56826259	0.00658
ko03410	Base excision repair	4.097042944	IPM_T2	2.87509248	0.00658
ko05012	Parkinson disease	0.595541311	IPM_T2	2.51817228	0.00567
ko05150	Staphylococcus aureus infection	3.366404589	IPM_T2	2.79846607	0.00658
ko00550	Peptidoglycan biosynthesis	4.375436009	IPM_T2	3.16020094	0.00658
ko03030	DNA replication	4.183480756	IPM_T2	2.9793101	0.00658
ko00785	Lipoic acid metabolism	4.17536769	IPM_T2	3.42511562	0.00658

Abbreviations, ETP, etapenem; IPM, imipenem/cilastatin; MEM, meropenem; T2 is the time point on the day after carbapnem admisnistation (day -1).

Table S3. Compared with the saline group, the carbapenems caused an increase in metabolic pathways.

Biomarker_KO_ID	Pathway Name	the logarithm value	increased groups	LDA_values	P_values
ko00030	Pentose phosphate pathway	4.329861385	IPM_T2	3.20175091	0.00658
ko00230	Purine metabolism	4.030304433	IPM_T2	2.79698635	0.00658
ko05111	Biofilm formation - Vibrio cholerae	3.332286277	IPM_T2	2.28950069	0.00658
ko00900	Terpenoid backbone biosynthesis	4.244388843	IPM_T2	3.14640014	0.00658
ko00471	D-Glutamine and D-glutamate metabolism	4.409656567	IPM_T2	2.84965401	0.00658
ko00473	D-Alanine metabolism	4.352652831	IPM_T2	3.0519913	0.00658
ko00240	Pyrimidine metabolism	4.136684126	IPM_T2	2.87488056	0.00658
ko01055	Biosynthesis of vancomycin group antibiotics	4.419794078	IPM_T2	3.15978373	0.00658
ko03050	Proteasome	1.510399599	IPM_T2	2.0283925	0.00658
ko00680	Methane metabolism	3.692910406	MEM_T2	2.20760235	0.00658
ko00010	Glycolysis / Gluconeogenesis	4.245273924	MEM_T2	3.0752064	0.00658
ko02060	Phosphotransferase system (PTS)	4.273542101	MEM_T2	3.4867045	0.00658
ko00300	Lysine biosynthesis	4.305297631	MEM_T2	3.1894832	0.00658
ko00260	Glycine, serine and threonine metabolism	4.017520479	MEM_T2	2.41882948	0.00658
ko00061	Fatty acid biosynthesis	4.282063172	MEM_T2	2.92310813	0.00658
ko00500	Starch and sucrose metabolism	4.043757764	MEM_T2	2.33676891	0.00658
ko00620	Pyruvate metabolism	4.140357174	MEM_T2	2.88637242	0.00658
ko00270	Cysteine and methionine metabolism	4.125767593	MEM_T2	2.65329262	0.00658
ko00520	Amino sugar and nucleotide sugar metabolism	4.19131665	MEM_T2	3.05408471	0.00658
ko00051	Fructose and mannose metabolism	4.180631347	MEM_T2	2.91482716	0.00658
ko00640	Propanoate metabolism	3.877610494	MEM_T2	2.43167254	0.00658
ko03410	Base excision repair	4.060637096	MEM_T2	2.32631616	0.00658
ko00550	Peptidoglycan biosynthesis	4.34503788	MEM_T2	2.86171245	0.00658
ko00030	Pentose phosphate pathway	4.302623975	MEM_T2	2.79261668	0.00658
ko00230	Purine metabolism	3.997991795	MEM_T2	2.1661217	0.00658

Abbreviations, ETP, etapenem; IPM, imipenem/cilastatin; MEM, meropenem; T2 is the time point on the day after carbapnem admisnistation (day -1).
