# Supplementary material for: The impact of three carbapenems at a single-day dose on intestinal colonization resistance against carbapenem-resistant Klebsiella pneumoniae
Source: mSphere. 2023 Nov 27;8(6):e00479-23. doi: 10.1128/msphere.00479-23 (PMC10732052; doi:10.1128/msphere.00479-23)
Supplement: Table S4 — Compared with the saline group, the carbapenems caused an decrease in metabolic pathways. [file msphere.00479-23-s0004.pdf]

Table S4. Compared with the saline group, the carbapenems caused an decrease in metabolic pathways.

Biomarker_ KO_ID	Pathway Name	the logarithm value	decreased groups	LDA_values	P_values
ko00740	Riboflavin metabolism	3.701204345	ETP_T2	2.6479912	0.00658
ko00510	N-Glycan biosynthesis	2.762662895	ETP_T2	2.3079819	0.00658
ko00061	Fatty acid biosynthesis	4.23988364	ETP_T2	3.1554656	0.00658
ko00100	Steroid biosynthesis	1.878520327	ETP_T2	2.306537	0.00658
ko03013	RNA transport	2.827325994	ETP_T2	2.237329	0.00658
ko00720	Carbon fixation pathways in prokaryotes	3.978722425	ETP_T2	2.7029091	0.00658
ko00790	Folate biosynthesis	3.943452554	ETP_T2	2.8542932	0.00658
ko00020	Citrate cycle (TCA cycle)	3.887216023	ETP_T2	2.5037118	0.00658
ko02030	Bacterial chemotaxis	3.982713156	ETP_T2	3.2932791	0.00658
ko00460	Cyanoamino acid metabolism	3.90627279	ETP_T2	3.5634959	0.00244
ko00780	Biotin metabolism	4.039452355	ETP_T2	3.275087	0.00658
ko04122	Sulfur relay system	3.951649904	ETP_T2	2.6366107	0.00658
ko03020	RNA polymerase	4.005749075	ETP_T2	2.9360201	0.00658
ko00860	Porphyrin and chlorophyll metabolism	3.539213218	ETP_T2	2.7505533	0.00658
ko00906	Carotenoid biosynthesis	1.773901047	ETP_T2	2.4209787	0.00658
ko00440	Phosphonate and phosphinate metabolism	3.276236054	IPM_T2	2.2846981	0.00658
ko00250	Alanine, aspartate and glutamate metabolism	4.238059995	IPM_T2	3.0151019	0.00658
ko00740	Riboflavin metabolism	3.701204345	IPM_T2	2.9546922	0.00658
ko04141	Protein processing in endoplasmic reticulum	2.632469775	IPM_T2	2.2036288	0.00658
ko00511	Other glycan degradation	4.084341947	IPM_T2	3.5456799	0.00658
ko00510	N-Glycan biosynthesis	2.762662895	IPM_T2	2.4153849	0.00658
ko04974	Protein digestion and absorption	2.318985082	IPM_T2	2.2003932	0.00658
ko00750	Vitamin B6 metabolism	3.929271839	IPM_T2	2.7572302	0.00658
ko02040	Flagellar assembly	3.747971155	IPM_T2	3.2180199	0.00658
ko05120	Epithelial cell signaling in Helicobacter pylori infection	3.088266887	IPM_T2	2.0166914	0.00658
ko00312	wingless-type MMTV integration site family, member 3	3.339730009	IPM_T2	2.8946873	0.00658
ko00531	Glycosaminoglycan degradation	3.777889332	IPM_T2	3.3482587	0.00658
ko00633	Nitrotoluene degradation	3.238425068	IPM_T2	2.5967714	0.00658
ko00040	Pentose and glucuronate interconversions	3.780701354	IPM_T2	2.7132641	0.00658

Abbreviations, ETP, etapenem; IPM, imipenem/cilastatin; MEM, meropenem; T2 is the time point on the day after carbapnem admisnistation (day -1).

Table S4. Compared with the saline group, the carbapenems caused an decrease in metabolic pathways.

Biomarker_ KO_ID	Pathway Name	the logarithm value	decreased groups	LDA_values	P_values
ko04146	Peroxisome	3.209013202	IPM_T2	2.0067566	0.00658
ko00600	Sphingolipid metabolism	3.600261444	IPM_T2	3.1320959	0.00658
ko00521	Streptomycin biosynthesis	4.196218486	IPM_T2	2.836736	0.00658
ko05146	Amoebiasis	1.620669813	IPM_T2	2.6180172	0.00658
ko00400	Phenylalanine, tyrosine and tryptophan biosynthesis	4.018237183	IPM_T2	3.0309086	0.00658
ko00720	Carbon fixation pathways in prokaryotes	3.978722425	IPM_T2	2.833109	0.00658
ko00960	Tropane, piperidine and pyridine alkaloid biosynthesis	3.431990391	IPM_T2	2.6948015	0.00658
ko00290	Valine, leucine and isoleucine biosynthesis	4.29835705	IPM_T2	3.0730408	0.00658
ko00410	beta-Alanine metabolism	3.5739967	IPM_T2	3.0264157	0.00658
ko00790	Folate biosynthesis	3.943452554	IPM_T2	3.0900338	0.00658
ko00020	Citrate cycle (TCA cycle)	3.887216023	IPM_T2	2.4660971	0.00658
ko00710	Carbon fixation in photosynthetic organisms	4.182963284	IPM_T2	2.4615419	0.00658
ko00340	Histidine metabolism	4.026131523	IPM_T2	3.3123991	0.00658
ko02030	Bacterial chemotaxis	3.982713156	IPM_T2	3.5161439	0.00658
ko04621	NOD-like receptor signaling pathway	2.770636083	IPM_T2	2.237354	0.00658
ko00460	Cyanoamino acid metabolism	3.90627279	IPM_T2	3.4427716	0.00484
ko00780	Biotin metabolism	4.039452355	IPM_T2	3.3957398	0.00658
ko00540	Lipopolysaccharide biosynthesis	3.635386772	IPM_T2	3.1879565	0.00658
ko00660	C5-Branched dibasic acid metabolism	4.151257294	IPM_T2	3.2723008	0.00658
ko03020	RNA polymerase	4.005749075	IPM_T2	2.925732	0.00658
ko00140	Steroid hormone biosynthesis	2.407720126	IPM_T2	2.1929626	0.00658
ko00860	Porphyrin and chlorophyll metabolism	3.539213218	IPM_T2	2.9875092	0.00658
ko00330	Arginine and proline metabolism	3.816137955	IPM_T2	2.5266522	0.00658
ko00906	Carotenoid biosynthesis	1.773901047	IPM_T2	2.0143375	0.00658
ko04210	Apoptosis	2.389406598	IPM_T2	2.151375	0.00658
ko00770	Pantothenate and CoA biosynthesis	4.206209154	IPM_T2	2.9914914	0.00658
ko00740	Riboflavin metabolism	3.70119086	MEM_T2	2.7450118	0.00658
ko00310	Lysine degradation	3.19774886	MEM_T2	2.25069	0.00658
ko00511	Other glycan degradation	4.084338714	MEM_T2	2.9807001	0.00658
ko00510	N-Glycan biosynthesis	2.762604693	MEM_T2	2.1741168	0.00658

Abbreviations, ETP, etapenem; IPM, imipenem/cilastatin; MEM, meropenem; T2 is the time point on the day after carbapnem admisnistation (day -1).

Table S4. Compared with the saline group, the carbapenems caused an decrease in metabolic pathways.

Biomarker_ KO_ID	Pathway Name	the logarithm value	decreased groups	LDA_values	P_values
ko04974	Protein digestion and absorption	2.318717802	MEM_T2	2.0662004	0.00658
ko03070	Bacterial secretion system	3.838021397	MEM_T2	2.6131156	0.00658
ko01053	iosynthesis of siderophore group nonribosomal peptide	2.460607395	MEM_T2	2.1308202	0.00658
ko00100	Steroid biosynthesis	1.878709259	MEM_T2	2.1918285	0.00658
ko00071	Fatty acid degradation	3.525650306	MEM_T2	2.458767	0.00658
ko00360	Phenylalanine metabolism	3.304734099	MEM_T2	2.1387805	0.00658
ko00130	Ubiquinone and other terpenoid-quinone biosynthesis	3.430964851	MEM_T2	2.7552064	0.00658
ko00040	Pentose and glucuronate interconversions	3.780700383	MEM_T2	2.58456	0.00658
ko00281	Geraniol degradation	3.013791392	MEM_T2	2.4129182	0.00658
ko04146	Peroxisome	3.209002387	MEM_T2	2.3168266	0.00658
ko00600	Sphingolipid metabolism	3.600272084	MEM_T2	2.7140661	0.00658
ko05143	African trypanosomiasis	1.428983296	MEM_T2	2.1675181	0.00658
ko05146	Amoebiasis	1.620912443	MEM_T2	2.5846532	0.00658
ko00350	Tyrosine metabolism	3.370454983	MEM_T2	2.3671717	0.00658
ko00720	Carbon fixation pathways in prokaryotes	3.978716873	MEM_T2	2.3798747	0.00658
ko00960	Tropane, piperidine and pyridine alkaloid biosynthesis	3.431982871	MEM_T2	2.4380626	0.00658
ko00790	Folate biosynthesis	3.943453565	MEM_T2	2.8716391	0.00658
ko00020	Citrate cycle (TCA cycle)	3.887217278	MEM_T2	2.7205925	0.00658
ko00460	Cyanoamino acid metabolism	3.906274903	MEM_T2	2.8669121	0.00658
ko00780	Biotin metabolism	4.039454664	MEM_T2	3.1697966	0.00658
ko00540	Lipopolysaccharide biosynthesis	3.635395929	MEM_T2	3.0387738	0.00658
ko03020	RNA polymerase	4.005745282	MEM_T2	2.4624913	0.00658
ko00140	Steroid hormone biosynthesis	2.407531545	MEM_T2	2.0271086	0.00658
ko00860	Porphyrin and chlorophyll metabolism	3.539203792	MEM_T2	2.7713347	0.00658
ko00472	D-Arginine and D-ornithine metabolism	3.108712429	MEM_T2	2.5873606	0.00658
ko04210	Apoptosis	2.38954588	MEM_T2	2.0004572	0.00658
ko03050	Proteasome	0.89186677	MEM_T2	2.2919111	0.00658
ko00930	Caprolactam degradation	2.737930261	MEM_T2	2.1234972	0.00658

Abbreviations, ETP, etapenem; IPM, imipenem/cilastatin; MEM, meropenem; T2 is the time point on the day after carbapnem admisnistation (day -1).
