# Supplementary material for: The impact of three carbapenems at a single-day dose on intestinal colonization resistance against carbapenem-resistant Klebsiella pneumoniae
Source: mSphere. 2023 Nov 27;8(6):e00479-23. doi: 10.1128/msphere.00479-23 (PMC10732052; doi:10.1128/msphere.00479-23)
Supplement: Table S5 — The metabolic pathways altered by the meropenem and imipenem groups compared to ertapenem. [file msphere.00479-23-s0005.pdf]

Table S5. The metabolic pathways altered by the meropenem and imipenem groups compared to ertapenem.

Biomarker _KO_ID	Pathway Name	the logarithm value	increased groups	LDA_values	P_values
ko00052	Galactose metabolism	4.124050976	ETP_T2	2.94893962	0.0066
ko00360	Phenylalanine metabolism	3.417233448	ETP_T2	2.53631777	0.0066
ko00450	Selenocompound metabolism	4.02719077	ETP_T2	2.46656422	0.0066
ko00472	D-Arginine and D-ornithine metabolism	3.612869296	ETP_T2	3.17324729	0.0066
ko00760	Nicotinate and nicotinamide metabolism	4.032248815	ETP_T2	2.49965407	0.0066
ko01053	Biosynthesis of siderophore group nonribosomal peptides	2.650899456	ETP_T2	2.09561002	0.0066
ko05143	African trypanosomiasis	2.279165861	ETP_T2	2.17738485	0.0066

Abbreviations, ETP, etapenem; IPM, imipenem/cilastatin; MEM, meropenem; T2 is the time point on the day after carbapnem admisnistation (day -1).

Table S5. The metabolic pathways altered by the meropenem and imipenem groups compared to ertapenem.

Biomarker _ KO_ID	Pathway Name	the logarithm value	decreased groups	LDA_values	P_values
ko00061	Fatty acid biosynthesis	4.23988364	ETP_T2	3.15546559	0.0066
ko03013	RNA transport	2.827325994	ETP_T2	2.23732905	0.0066
ko04122	Sulfur relay system	3.951649904	ETP_T2	2.63661075	0.0066

Abbreviations, ETP, etapenem; IPM, imipenem/cilastatin; MEM, meropenem; T2 is the time point on the day after carbapnem admisnistation (day -1).
